# Effect of Prophylactic Use of Intranasal Oil Formulations in the Hamster Model of COVID-19

**DOI:** 10.3389/fphar.2021.746729

**Published:** 2021-10-14

**Authors:** Zaigham Abbas Rizvi, Manas Ranjan Tripathy, Nishant Sharma, Sandeep Goswami, N Srikanth, J. L. N. Sastry, Shailendra Mani, Milan Surjit, Amit Awasthi, Madhu Dikshit

**Affiliations:** ^1^ Immuno-biology Laboratory, Infection and Immunology Centre, Translational Health Science and Technology Institute, NCR-Biotech Science Cluster, Faridabad, India; ^2^ Infection and Immunology Centre, Translational Health Science and Technology Institute, NCR-Biotech Science Cluster, Faridabad, India; ^3^ DG(I/C), Central Council for Ayurvedic Sciences, New Delhi, India; ^4^ CEO-National Medicinal Plants Board, Ministry of AYUSH, New Delhi, India; ^5^ Non-communicable Disease Centre, Translational Health Science and Technology Institute, NCR-Biotech Science Cluster, Faridabad, India

**Keywords:** COVID-19, intranasal, herbal, AYUSH, prophylactic

## Abstract

Severe acute respiratory syndrome coronavirus 2 (SARS-CoV2) infection initiates with viral entry in the upper respiratory tract, leading to coronavirus disease 2019 (COVID-19). Severe COVID-19 is characterized by pulmonary pathologies associated with respiratory failure. Thus, therapeutics aimed at inhibiting the entry of the virus or its internalization in the upper respiratory tract are of interest. Herein, we report the prophylactic application of two intranasal formulations provided by the National Medicinal Plant Board (NMPB), Anu oil and til tailya, in the hamster model of SARS-CoV-2 infection. Prophylactic intra-nasal instillation of these oil formulations exhibited reduced viral load in lungs and resulted in reduced body weight loss and lung-pneumonitis. In line with reduced viral load, histopathological analysis revealed a reduction in lung pathology in the Anu oil group as compared to the control infected group. However, the til tailya group did not show a significant reduction in lung pathology. Furthermore, molecular analysis using mRNA expression profiling indicated reduced expression of pro-inflammatory cytokine genes, including Th1 and Th17 cytokines for both the intranasal formulations as a result of decreased viral load. Together, the prophylactic intranasal application of Anu oil seems to be useful in limiting both viral load and severity in SARS-CoV2 infection in the hamster model.

## Introduction

Since the first report of Coronavirus Disease (COVID-19) in Wuhan in December 2019, a number of COVID-19 incidences have exploded around the globe leading it to be declared a pandemic by the WHO (Chen and Li, 2020; Wang et al., 2020) (https://www.ecdc.europa.eu/en/geographical-distribution-2019-ncov-cases). As of September 6, 2021, the total number of coronavirus infection incidences was 221,846,104 with around 4,586,516 deaths globally, with 441,075 mortalities in India alone. The majority of the coronavirus cases are asymptomatic and do not require aggressive treatment. However, an estimated 13.8% of the infected individuals are at risk of developing a severe form of COVID-19, which could be characterized by either one or all of the following COVID-19 symptoms: respiratory distress, high fever, loss of taste and smell, and diarrhea (Chen and Li, 2020; Wang et al., 2020). In addition, up to around 6% of COVID-19 cases end up with respiratory failure due to cytokine storm, cardiovascular complications, and multiple organ failure (https://www.who.int/emergencies/diseases/novel-coronavirus-2019) (Guan et al., 2020; Wang et al., 2020). As is known for other respiratory viruses, SARS-CoV2 initially infects the upper respiratory tract and then rapidly spreads to the lower respiratory tract (Chen and Li, 2020). During an active infection, the virus can be transmitted and spread from both symptomatic and asymptomatic individuals via respiratory droplets generated through coughing, sneezing, or hyperventilation via the airborne route (Gandhi et al., 2020; Guan et al., 2020).

Global health research has primarily focused on vaccine development against COVID-19, with active vaccination being the current strategy to protect COVID-19–related mortalities (Poland et al., 2020a; Dong et al., 2020). Given the emergence of new SARS-CoV2 variants, the protective efficacy of vaccines could be reduced; hence, therapeutics that may prevent viral entry, replication, and transmission are highly desirable. In line with this, pharmacological agents such as intranasal delivery of TLR2/6 agonist or lipopeptide agents, intranasal administration of neutralizing antibodies, and intranasal gene therapy are currently being explored as potential strategies to inhibit the host–pathogen interaction and limit the infection (Hassan et al., 2020; Boiardi and Stebbing, 2021; Ku et al., 2021; Kunzelmann, 2021; Proud et al., 2021; Vries et al., 2021). For example, intranasal corticosteroid spray for the recovery of the sense of smell is under clinical trials for COVID-19 patients (Abdelalim et al., 2021). Since pharmaceutical drugs may have many off-target effects, therapeutics based on herbal extracts have recently gained much attention (Matveeva et al., 2020; De Pellegrin et al., 2021; Jan et al., 2021; Li et al., 2021). Here, we evaluated the efficacy of two ayurvedic intranasal (herbal) oil formulations, Anu oil and til tailya (Duraipandi and Selvakumar, 2020), in hamster SARS-CoV2 challenge model.

Sesame oil (til tailya, TT) is the oil derived from a plant (*Sesamum indicum*) which is a classical ayurvedic medicine mentioned in Charak Samhita (https://niimh.nic.in/ebooks/ecaraka/). On the other hand, a classical ayurvedic medicine, Anu tailya was used by Maharishi Charak more than 5,000 years ago for therapeutic purposes. Anu oil consists of extracted oils from several important medicinal plants like nāgarmothā (*Cyperus scariosus*), jīvantī (*Leptadenia reticulata*), sweta candana (*Santalum album*), jala (*Pavonia odorata*), Pṛśniparṇī (*Uraria picta*), bela (*Aegle marmelos*), devdāru (*Cedrus deodara*), dāruharidrā (*Berberis aristata*), tejpatra (*Cinnamomum tamala*), dālacīnī (*Cinnamomum verum*), kamala keṣara (*Nelumbo nucifera*), sevya (*Chrysopogon zizanioides*), viḍañga (*Embelia ribes*), utpala (*Nymphaeanouchali*), anantmūla (*Hemidesmus indicus*), tila tailya (*Sesamum indicum*), muleṭhī (*Glycyrrhiza glabra*), plawa (*Cyperus platyphyllus*), agarū (*Aquilaria agallocha*), satāvarī (*Asparagus racemosus*), bṛhatī (*Solanum indicum*), kaṇṭakārī (*Solanum surattense*), surbhi (*Pluchea lanceolata*), sālaparṇī (*Desmodium gangeticum*), truṭi (*Elettaria* cardamomum), reṇukā (*Vitex agnus-castus*), and ajadugdha (Duraipandi and Selvakumar, 2020; see the enclosed supplement information). Here, we report that intranasal instillation of both til tailya and Anu oil limited the viral entry and replication in the lungs associated with SARS-CoV2 infection in hamsters. However, Anu oil but not til tailya was able to rescue the lung pneumonitis and injury partly due to suppression of inflammatory cytokine response.

## Materials and Methods

Sesame oil and Anu oil (a polyherbal medicine) used in the study were prepared as per pharmacopoeial standards and were provided by the National Medicinal Plant Board (NMPB) for the study.

### Animal Ethics and Biosafety Statement

6- to 9-week-old golden Syrian hamsters were acclimatized in biosafety level-2 (BSL-2) for 1 week and then infected in the animal BSL3 (ABSL-3) institutional facility. The animals were maintained under the 12-h light and dark cycle and fed a standard pellet diet and water ad libitum. All the experimental protocols involving the handling of virus culture, and animal infection were approved by RCGM, institutional biosafety, and IAEC Animal Ethics Committee (IAEC/THSTI/105).

### Virus Culture and Titration

SARS-related coronavirus 2, isolate USA-WA1/2020 virus was grown and titrated in vero E6 cell line cultured in Dulbecco’s modified Eagle medium (DMEM) complete media containing 4.5 g/L D-glucose, 100,000 U/L penicillin–streptomycin, 100 mg/L sodium pyruvate, 25 mM HEPES, and 2% FBS. The stocks of the virus were plaque purified at the THSTI IDRF facility inside ABSL3 following institutional biosafety guidelines.

### SARS-CoV2 Infection in Golden Syrian Hamster and Ayush Herbal Extracts Dosing Regime

6- to 9-week-old golden Syrian hamsters were procured from CDRI and quarantined for 1 week at the small animal facility (SAF), THST before starting the experiment. The animals were then randomly divided into five groups containing five animals/group, namely, uninfected (UI), infected (I), and two infected groups receiving til tailya (TT) or Anu oil (AO) as therapeutic interventions, respectively. One group received intranasal installation of Anu oil, while the other group received intranasal installation of til tailya (50 ul/nostril/day) starting 5 days before infection and continued till 4 days postinfection (DPI). On the day of the challenge, intranasal administration of Anu oil and til tailya was carried out 30 min before infection. On the day of the challenge, the animals were shifted to ABSL3. Intranasal infection with live SARS-CoV2 10^5^PFU/100 μl or with the DMEM mock control (for uninfected control group) was established with the help of a catheter under mild anesthetized by using ketamine (150 mg/kg) and xylazine (10 mg/kg) intraperitoneal injection inside the ABSL3 facility. All the experimental protocols involving the handling of virus culture and animal infection were approved by the RCGM, Institutional Biosafety and IAEC Animal Ethics Committee.

### Gross Clinical Parameters of SARS-CoV2 Infection

All infected animals were euthanized after 4 days post-infection at ABSL3. Changes in body weight were observed each day post-challenge and plotted as percent change in the body weight. Post-sacrifice, the lungs and spleen of the animals were excised and imaged for gross morphological changes. The left lower lobe of the lung was fixed in 10% formalin and used for histological analysis. The remaining part of the lung’s left lobe was homogenized in 2 ml TRIzol solution for viral load estimation. The spleen was homogenized in 2 ml of TRIzol solution. The TRIzol samples were stored immediately at −80°C until further use. Blood of the animals was drawn through direct heart puncture, and serum was isolated and stored at -80°C until further use.

### Viral Load

For viral load, estimated lungs were homogenized in TRIzol reagent (Invitrogen), and their supernatant was collected after centrifugation at 4,000 rpm for 15 min at 4°C. Thereafter, RNA was isolated by TRIzol–choloform method, and RNA yield was quantitated by nano-drop; 1 µg of total RNA was then reverse-transcribed to cDNA using the iScript cDNA synthesis kit (Biorad; #1708891) (Roche). Diluted cDNAs (1:5) were used for qPCR by using the KAPA SYBR^®^ FAST qPCR Master Mix (5X) Universal Kit (KK4600) on the Fast 7,500 Dx real-time PCR system (Applied Biosystems), and the results were analyzed with SDS2.1 software. In brief, 200 ng of RNA was used as a template for reverse transcription polymerase chain reaction (RT-PCR). The CDC-approved commercial kit was used for the SARS-CoV-2 N gene: 5′-GAC​CCC​AAA​ATC​AGC​GAA​AT-3′ (forward), 5′-TCT​GGT​TAC​TGC​CAG​TTG​AAT​CTG-3′ (reverse). The hypoxanthine–guanine phosphoribosyltransferase (HGPRT) gene was used as an endogenous control for normalization through quantitative RT-PCR. The relative expression of each gene was expressed as fold change and was calculated by subtracting the cycling threshold (Ct) value of hypoxanthine–guanine phosphoribosyltransferase (HGPRT-endogenous control gene) from the Ct value of the target gene (ΔCT). The fold change was then calculated according to the formula POWER(2,-ΔCT)*10,000 (Malik et al., 2017).

### qPCR

RNA from spleen samples was isolated as described earlier for the lung samples, and cDNA was prepared. Thereafter, the relative expression of each gene was expressed as fold change and was calculated by subtracting the cycling threshold (Ct) value of hypoxantine–guanine phosphoribosyltransferase (HGPRT-endogenous control gene) from the Ct value of target gene (ΔCT). Fold change was calculated according to the formula POWER(2,-ΔCT)*10,000 (Malik et al., 2017; Rizvi et al., 2021a). The list of the primers is provided as follows in Table 1

**TABLE 1:** Primer sequences.

Gene	Forward	Reverse
HGPRT	GAT​AGA​TCC​ACT​CCC​ATA​ACT​G	TAC​CTT​CAA​CAA​TCA​AGA​CAT​TC
Tryptase β2	TCG​CCA​CTG​TAT​CCC​CTG​AA	CTA​GGC​ACC​CTT​GAC​TTT​GC
Chymase	ATG​AAC​CAC​CCT​CGG​ACA​CT	AGA​AGG​GGG​CTT​TGC​ATT​CC
muc1	CGG​AAG​AAC​TAT​GGG​CAG​CT	GCC​ACT​ACT​GGG​TTG​GTG​TAA​G
Sftpd	TGA​GCA​TGA​CAG​ACG​TGG​AC	GGC​TTA​GAA​CTC​GCA​GAC​GA
Eotaxin	ATG​TGC​TCT​CAG​GTC​ATC​GC	TCC​TCA​GTT​GTC​CCC​ATC​CT
PAI-1	CCG​TGG​AAC​CAG​AAC​GAG​AT	ACC​AGA​ATG​AGG​CGT​GTC​AG
IFNy	TGT​TGC​TCT​GCC​TCA​CTC​AGG	AAG​ACG​AGG​TCC​CCT​CCA​TTC
TNFa	AGA​ATC​CGG​GCA​GGT​CTA​CT	TAT​CCC​GGC​AGC​TTG​TGT​TT
IL13	AAATGGCGGGTTCTGTGC	AAT​ATC​CTC​TGG​GTC​TTG​TAG​ATG​G
IL17 A	ATG​TCC​AAA​CAC​TGA​GGC​CAA	GCG​AAG​TGG​ATC​TGT​TGA​GGT
IL10	GGT​TGC​CAA​ACC​TTA​TCA​GAA ATG	TTC​ACC​TGT​TCC​ACA​GCC​TTG
IL6	GGA​CAA​TGA​CTA​TGT​GTT​GTT​AGA​A	AGG​CAA​ATT​TCC​CAA​TTG​TAT​CCA​G
CXCL10	TGG​AAA​TTA​TTC​CTG​CAA​GTC​A	GTG ATC GGC TTC TCT CTG GT

### Histology

The lung of the euthanized animals was fixed in 10% formalin solution and then embedded in paraffin. Sample embedded paraffin blocks were then cut into 3-µm fine sections and mounted on silicone-coated glass slides. The slides were then stained with hematoxylin and eosin dye, as previously described (Rizvi et al., 2018). Each stained sample was then analyzed and captured at ×40 magnification. Assessment for the histological score was carried out through blind scoring for each sample by a professional histologist.

### Statistical Analysis

Results from the experiments were analyzed and plotted by using GraphPad Prism 7.0 software. The graph for percent change in body weight, gene expression, and lung histology scores were compared and analyzed by using the Student t-test or one-way ANOVA, with *n* = 5 samples per group. The *p*-value of less than 0.05 and was considered as statistically significant.

## Results

### Prophylactic Use of Intranasal Instillation of Ayush Oil Formulations Prevents SARS-CoV2 Infection and Associated Gross Clinical Parameters

**FIGURE 1:**
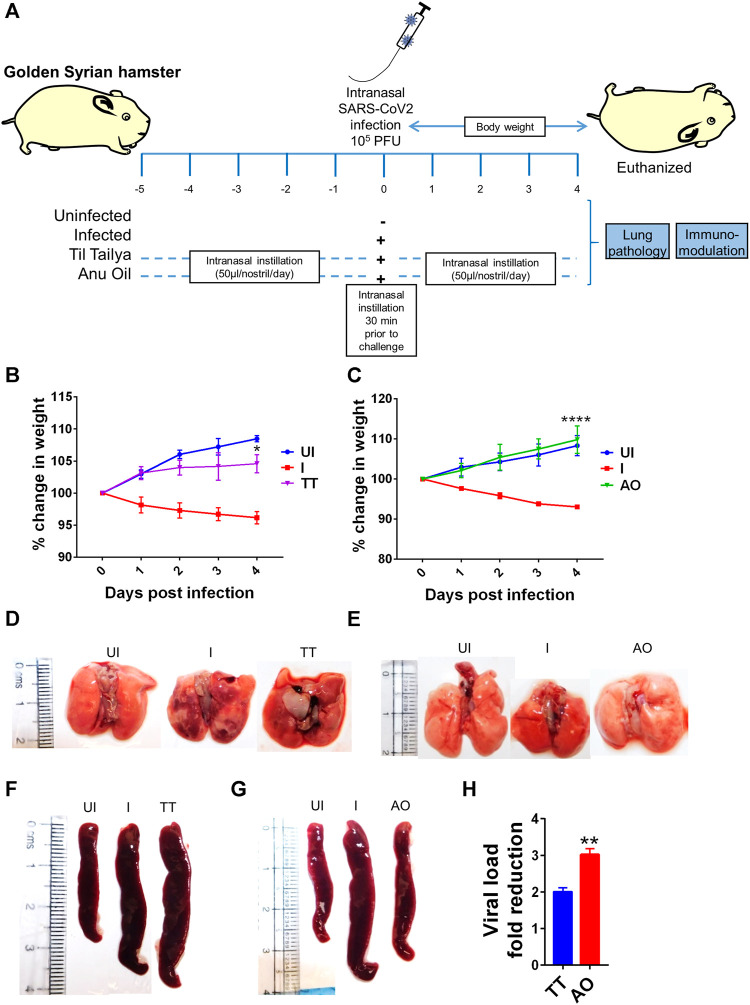
Effect of intranasal instillation of Anu oil and til tailya on gross clinical parameters and lung viral load in SARS-CoV2 infected hamsters. **(A)** Schematic outlines the study design. Prophylactic treatment regime was adopted for Anu oil (AO) and til tailya (TT) with each animal receiving (50 µl/nostril/day) intranasal instillation of Anu oil, til tailya, or mock control 5 days before challenge and then continued till after infection till end point (i.e., 4 days postinfection). One group was challenge and received live infection (I); the other group of animals was unchallenged healthy control (UI). On the day of challenge, the animals were given intranasal oil-instillation 30 min prior to challenge with SARS-CoV2. **(B and C)** Line graph showing mean % change in body weight post-infection ±standard error mean (SEM). **(D and E)** Images of the excised lungs showing gross morphology with pneumonitis region (dark red patches). **(F and G)** Images of excised spleen indicating changes in the spleen length. **(H)** Bar graph showing mean fold reduction in lung viral load ±SEM as compared to the infected **(I)** control. *p < 0.05, **p < 0.01, ****p < 0.0001 (*t*-test).

SARS-CoV2 infection in hamsters peaks in 4–5 days and is characterized by a reduction in body weight and appearance of pneumonitis in the lungs and splenomegaly (Imai et al., 2020; Sia et al., 2020; Rizvi et al., 2021b; Chan et al., 2020). These defined gross clinical parameters were recorded for all the groups, that is, uninfected control (UI), infected control (I), and infected hamsters receiving either Til tailya (TT) or Anu oil (AO) intranasal formulations (50 µl/nostril/day). Treatment of TT and AO was started 5 days before SARS-CoV2 live virus challenge and was continued till the end of the experiment, that is, 4 days postinfection (dpi), as presented schematically in the study design (Figure 1A). In line with the earlier published reports, a decrease in the body weight of SARS-CoV2–infected hamsters was observed with 5–8% body weight reduction on 4 days dpi. Hamsters receiving TT or AO, before SARS-CoV2 infection, did not lose body weight as observed in the SARS-CoV2–infected group (Figures 1B,C). SARS-CoV2 infection in the hamster model is characterized by lung inflammation, pneumonitis, and cytokines release (Afrin et al., 2020; Chen and Li, 2020; Moore and June 2020). To further understand the lung-associated pathologies, gross morphological changes of the excised lungs were compared between healthy, SARS-CoV2–infected, and SARS-CoV2–infected plus oil formulated groups. There was a reduction in the regions of pneumonitis in the excised lungs of the AO group, but not the TT group, as compared to infection control (Figures 1D,E). As reported earlier, splenomegaly is one of the critical parameters indicating active infection (Rizvi et al., 2021b). Thus, we tested the splenomegaly between different groups and found that AO, but not TT, showed inhibition in splenomegaly as compared to the SARS-CoV2–infected hamsters (Figures 1F,G). We also evaluated the lung viral load at four dpi and calculated the fold reduction in viral load in AO- and TT-treated groups as compared to the SARS-CoV2–infected groups. Our data indicate that compared to the SARS-CoV2–infected group, viral loads in AO- and TT-treated groups were ∼3- and ∼2-fold less, respectively (Figure 1H). Together, these data indicated that prophylactic use of intranasal instillation of TT and AO resulted in decreased lung viral load with the AO group, showing better protection in gross clinical parameters.

### Prophylactic Use of Anu Oil Reduces SARS-CoV2–Induced Lung Pathology in Hamsters

**FIGURE 2:**
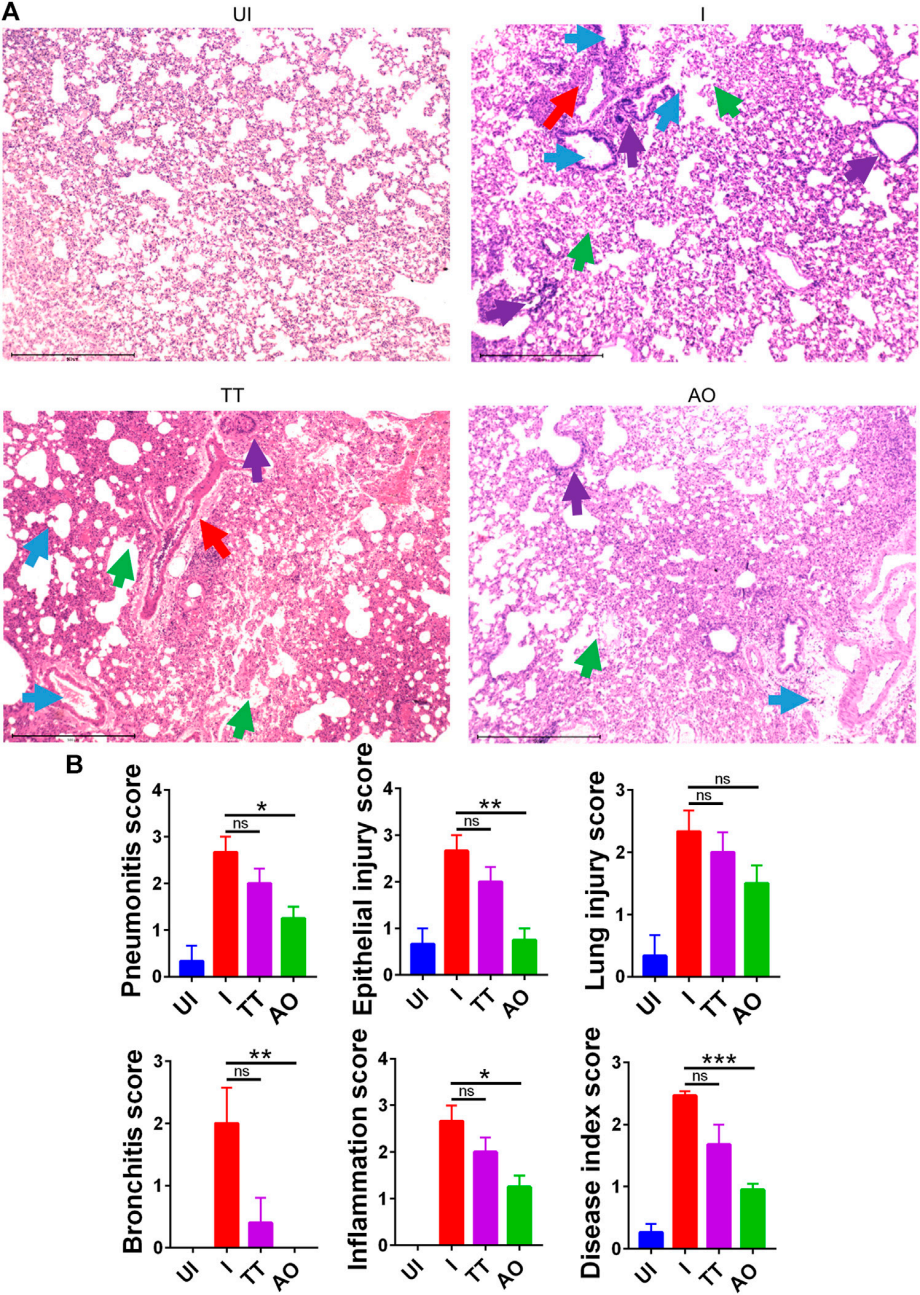
H&E–stained lung sections showing histopathology and its assessment. **(A)** images of H&E-stained lungs at ×40 magnification showing regions of pneumonitis (blue arrow), bronchitis (red arrow), epithelial injury (green arrow), and inflammation (purple arrow) along with their **(B)** histological score for pneumonitis, inflammation, lung injury, alveolar epithelial cells, bronchitis, and overall disease score for different groups UI, I, TT, and AO on day 4 postinfection. *p < 0.05, **p < 0.01, ***p < 0.001 (one-way ANOVA).

Since gross clinical parameters and lung viral load data suggested protection from SARS-CoV2 infection in AO and TT groups, we set out to study the mitigation of pulmonary pathologies such as lung injury, alveolar epithelial injury, bronchitis, pneumonitis, and inflammation using histological analysis (Bao et al., 2020; Lee et al., 2020; Leng et al., 2020; Rizvi et al., 2021b). Hematoxylin and eosin (H and E)–stained lung data showed a reduction in alveolar epithelial injury, inflammation, and pneumonitis in SARS-CoV2–infected AO-treated hamsters as compared to SARS-CoV2–infected hamsters. There was no sign of bronchitis in SARS-CoV2–infected AO-treated hamsters with overall significant mitigation in the disease score as compared to SARS-CoV2–infected hamsters (Figures 2A,B). Hamsters treated with TT however showed little or no improvement in lung injury and overall disease score as compared to the infected control (Figures 2A,B). Together, lung pathology associated with SARS-CoV2 was found to be resolved in the AO group but not in the TT group, with around 1.5-fold rescue in disease index in the AO group as compared to the infected control group.

### Prophylactic Use of Anu Oil Prevent Lung Injury in Hamsters Associated With SARS-CoV2 Infection

**FIGURE 3:**
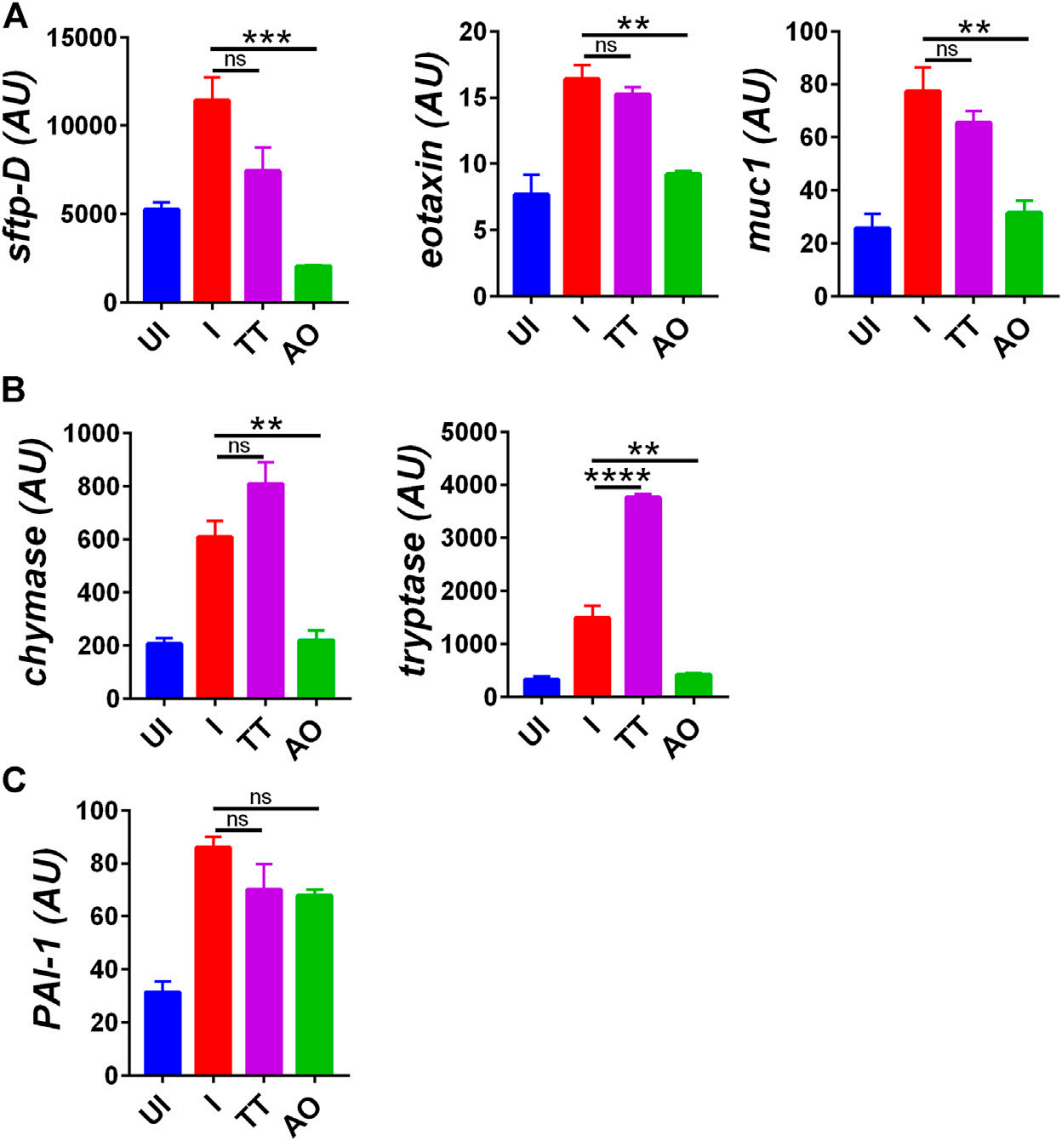
Changes in mRNA expression of genes involved in lung injury upon AO or TT intranasal administration in hamsters infected with SARS-CoV2. Relative mRNA expression profiling was carried out in UI, I, TT, and AO lung samples for **(A)** lung injury genes **(B)** mast cell activation markers **(C)** thrombosis factor. Mean ± SEM. **p < 0.01, ***p < 0.001, ****p < 0.0001 (one-way ANOVA).

Lung histology data indicated overall improvment in histological changes of SARS-CoV2–infected AO-treated groups as compared to SARS-CoV2–infected group. These data compelled us to explore the mechanism involved in the rescue of lung pathologies. Injury to the pulmonary region is characterized by elevated expression of surfactant D (sftp-D), increased mucus (muc-1) secretion, and increased expression of eotaxin, which promotes infiltration of granulocytes and mast cells and an increased risk of pulmonary thrombosis as seen in COVID-19 patients (Crouch, 2000; Guo et al., 2001; Prabhakaran et al., 2003; Xie et al., 2017; Chatterjee et al., 2020). We observed elevated levels of sftp-D, muc-1, eotaxin, muc-1, chymase, tryptase, and plasmonigen activator inhibitor-I (PAI-1: a key factor for lung fibrosis) in the lungs of infected hamsters (Figures 3A–C). However, prophylactic intranasal use of AO in SARS-CoV2–infected hamster significantly reduced the mRNA expression of these lung injury genes and genes that are required for chemotaxis of granulocytes and function of mast cells (Figures 3A,B). Prophylactic use of AO in SARS-CoV2–infected hamsters did not reduce PAI-1 expression (Figure 3C). In contrast to AO, TT data showed no reduction in lung injury as compared to infected control (Figure 3A). Surprisingly, there was a marked increase in mast cell markers in the TT group as compared to the infected control (Figure 3B). Overall, consistent with our lung histology data, a profound reduction in the expression of lung injury genes and mast cell markers as compared to the infected control was observed in AO-treated groups.

### AO and TT Treatment Inhibits the Expression of SARS-CoV2–Induced Pro-Inflammatory Cytokines

**FIGURE 4:**
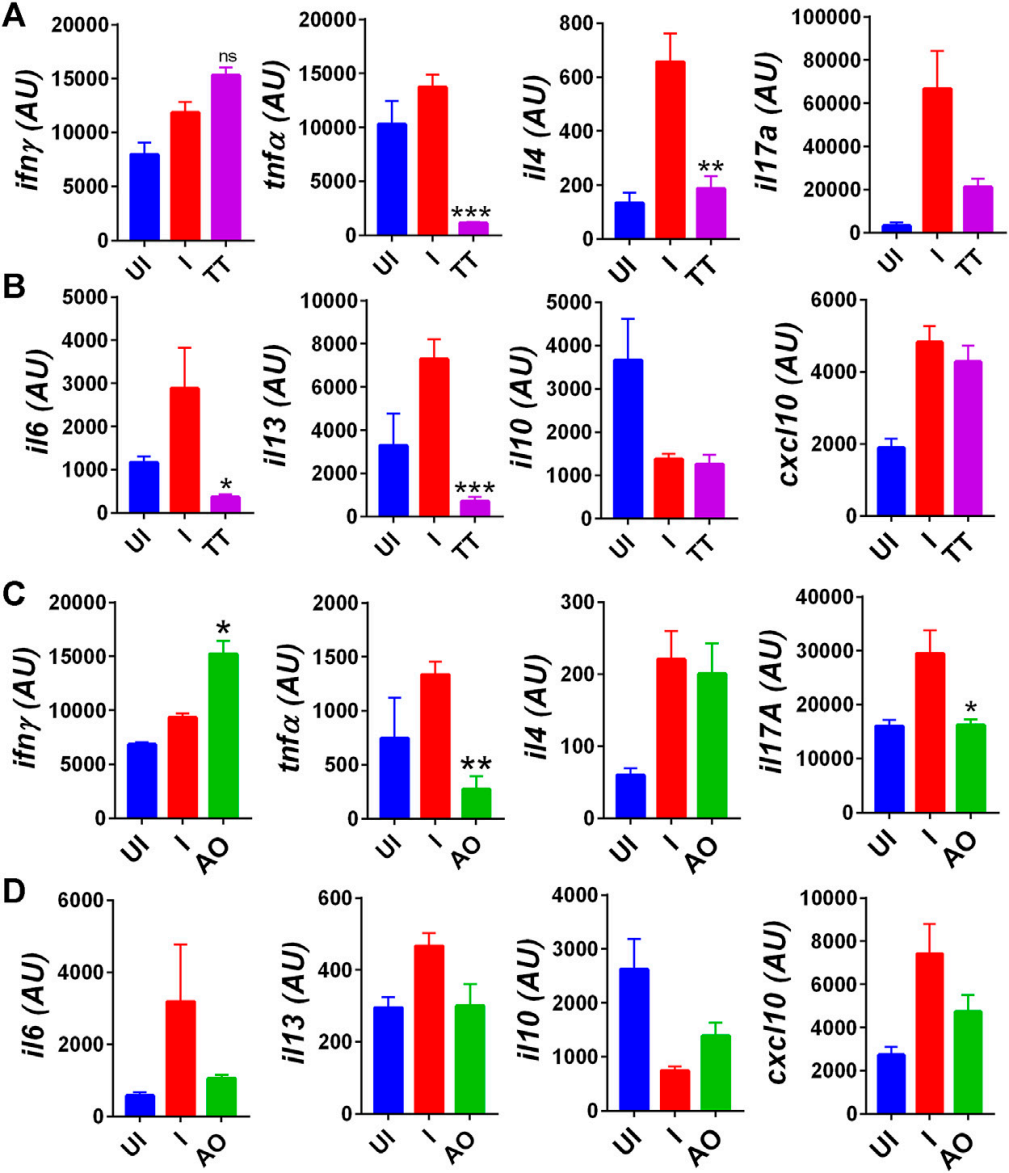
Immuno-modulatory effect of TT and AO on cytokine expression in splenocytes. Relative mRNA expression profiling was carried out in UI, I, TT, and AO splenocytes samples for **(A)** T helper cell cytokines for TT samples. **(B)** pro-inflammatory and anti-inflammatory cytokines for TT samples. **(C)** T helper cell cytokines for AO samples. **(D)** pro-inflammatory and anti-inflammatory cytokines for AO samples. Bar graph showing mean ± SEM. ns = non-significant **p < 0.05 **p < 0.01, ***p < 0.001, ****p < 0.0001 (one-way ANOVA).

COVID-19–related respiratory distress is associated with inflammation in the lungs. The increase in lung inflammation is characterized by the secretion of pro-inflammatory cytokines in COVID-19 patients (Iwasaki and Yang, 2020; Mathew et al., 2020; Moore and June 2020; Verity et al., 2020). Cytokine expression data from splenocytes indicate elevated expression of Th1 cytokines (IFNγ and TNFα), Th2 cytokine (IL-4, IL-13), Th17 cytokine (IL-17 A), and various other pro-inflammatory cytokine-like IL-6 and IL-13 in SARS-CoV2–infected group as compared to uninfected hamsters (Figures 4A–D). However, there was not much change observed in anti-inflammatory cytokine IL-10 expression as compared to the challenge control group. Prophylactic intranasal installation of AO and TT in SARS-CoV2–infected hamsters resulted in the reduction in Th1 and Th17 cell cytokines, together with pro-inflammatory cytokine expression (Figures 4A,B,D). However, surprisingly, only TT but not AO was able to reduce the Th2 cytokine gene expression (Figures 4A,C). Furthermore, we found an elevated IFN-gamma secretion in both AO- and TT-treated animals (Figures 4A,C). Since C-X-C motif chemokine ligand 10 (CXCL10), a chemoattractant, is an important mediator of IFN-gamma response and is secreted by various immune cells, we evaluated the mRNA expression of CXCL10 from treated (AO/TT) vs challenge (I) samples (Zhang et al., 2020). As compared to the challenge control group, neither the AO nor TT group did not show significant changes (Figures 4B,D). Together, we show that AO and TT reduced the pro-inflammatory cytokines.

#### Anu Oil Intranasal Formulation Shows More Protective Efficacy Against SARS-CoV2 Infection in Hamsters as Compared to Til Tailya Intranasal Formulation

**FIGURE 5:**
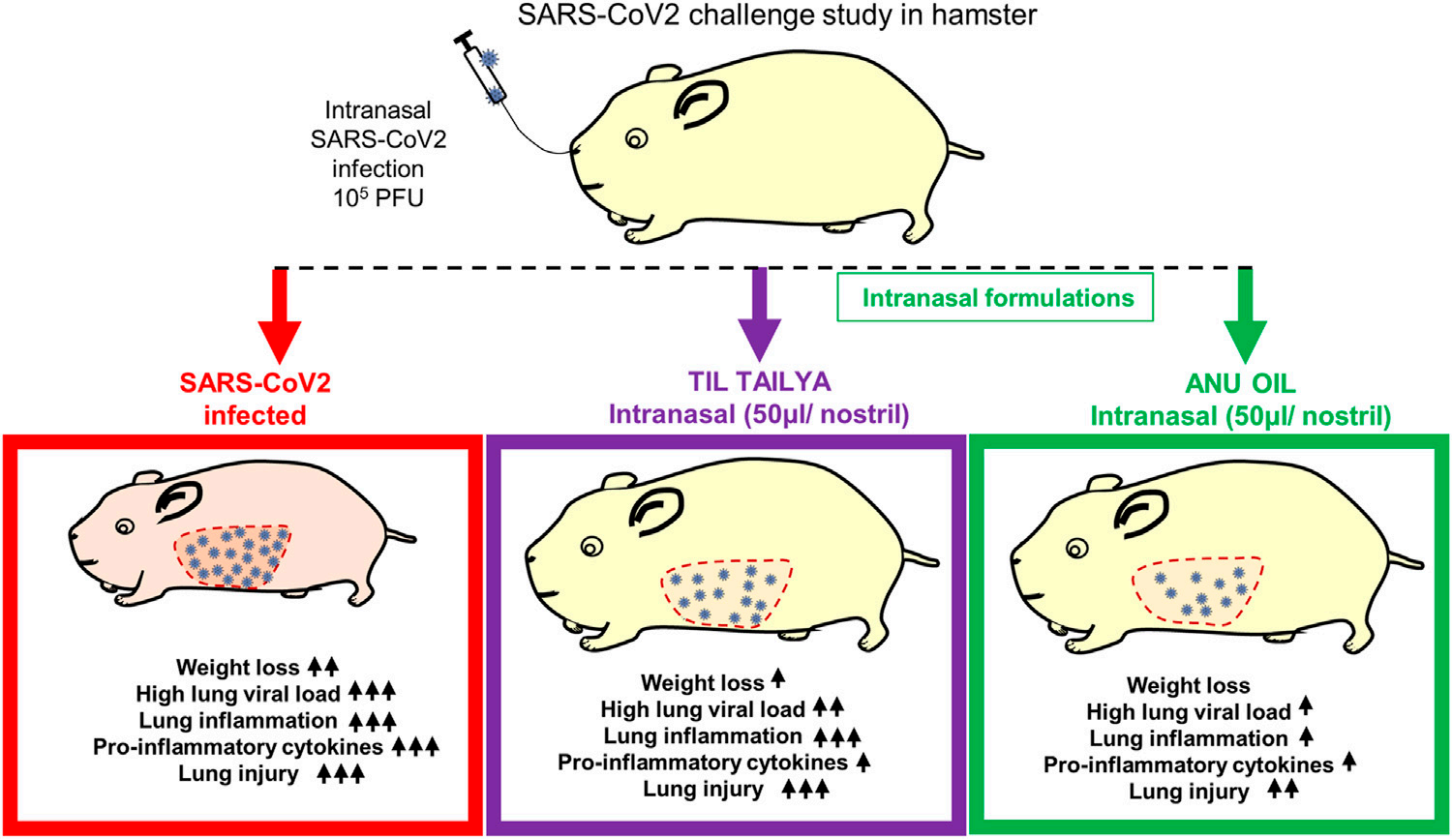
Summary representing the important findings of the study. Diagrammatic representation of the effect of pretreatment of intranasal til tailya and Anu oil against SARS-CoV2–associated pathologies in golden Syrian hamster. ns = non-significant.

We summarize the finding of our study and provide the first evidence that intranasal formulation such as Anu oil and til tailya limits viral entry and replication. However, only Anu oil but not til tailya was effective in reducing the SARS-CoV2–associated pulmonary pathologies and lung injuries, even though both AO and TT were effective in reducing the pro-inflammatory cytokine response in hamsters (Figure 5).

## Discussion

Advances in vaccine development against SARS-CoV2 Wuhan strain and aggressive vaccination strategy have significantly reduced the SARS-CoV2 infection and COVID-19 deaths worldwide (Dong et al., 2020; Poland et al., 2020a, 2020b). However, the recent rise in variant strains that show high transmission and disease severity is of concern, primarily, due to the reduced efficacy of virus neutralization in vaccinated individuals for some variant strains (Garcia-Beltran et al., 2021; Planas et al., 2021; Supasa et al., 2021). Therefore, therapeutic options are paramount in combating COVID-19.

Although several repurposed drugs are currently being used for the treatment of COVID-19 patients, they have limited efficacy. Herbal medicines or medicines derived from herbal extracts offer a safer alternative therapy due to their prolonged human use, acceptability with lesser side effects (Matveeva et al., 2020; Jan et al., 2021; Li et al., 2021). Recently, Chinese traditional medicine has gained popularity due to its antiviral efficacy in *in vitro* and animal models of SARS-CoV2 (Ling, 2020; Xiong et al., 2020; Yang et al., 2020; Jan et al., 2021; Lee et al., 2021). In India, going back to more than 3,000 years, ayurvedic medicines are considered useful for both lifestyle disorders and infectious conditions. The word ayurveda is derived from two Sanskrit words ayur (life) and veda (knowledge) (Subrahmanya et al., 2013; Banerjee et al., 2020; Girija and Sivan, 2020; Golechha, 2020; Joshi and Puthiyedath, 2020; Rastogi et al., 2020). In the present study, we investigated antiviral activity of two oil formulations, namely, til tailya and Anu oil against SARS-CoV2. Due to immiscibility of these oil formulations with culture medium, it was not possible to test them *in vitro* for their antiviral activity using VeroE6 cell line.

Therefore, in the current study, we describe the efficacy of prophylactic use of two intranasal ayurvedic oil formulations using the hamster model for SARS-CoV2 challenge. Hamsters are one of the best small animal models for SARS-CoV2 infection which mimics the viral entry and replication of the upper and the lower respiratory tract of humans (Sia et al., 2020; Rizvi et al., 2021b). Hamsters receiving Anu oil or til tailya intranasally before SARS-CoV2 infection showed reduced weight loss. Lung histology data corroborated with the gross parameter findings showed lesser SARS-CoV2–related histopathology in Anu oil–treated hamsters, while there was little or no protection in the til tailya group. It has been shown earlier that even low dose of intranasal SARS-CoV2 infection in hamsters could result in pneumonitis and lung pathologies (Rizvi et al., 2021b). It might be possible that even though both til tailya and Anu oil could limit the entry of SARS-CoV2 and replication, only Anu oil–treated group exhibited a greater reduction in the lung viral load (around three folds). Expectedly, the Anu oil group also showed lesser lung injury than the til tailya group. Pulmonary damage and pneumonitis have been reported as the major causes of respiratory failure in patients suffering from a severe form of SARS-CoV2 infection (Moore and June 2020). Prophylactic use of Anu oil showed significant reduction in the overall disease index as compared to the infected control, suggesting some degree of protection against SARS-CoV2–induced lung injury and pathology. The reagents and antibodies specific to hamster CD molecules and signaling proteins were commercially not available, thus limiting the scope of finding the molecular mechanisms and cellular characterization of the protective response. We therefore carried out mRNA expression profiling to assess the role of cytokines and chemokines involved in the inflammatory response and lung injury parameters. mRNA expression data suggest that Anu oil intervention also reduced the expression of lung injury markers and lung inflammation, indicating that Anu oil was able to protect against the pulmonary damage caused by SARS-CoV2 infection. Finally, we studied the expression of cytokines to understand if intranasal formulation could help prevent the inflammatory cytokine response within the lung. Interestingly, both Anu oil and til tailya were able to limit the expression of pro-inflammatory cytokines as compared to the infected hamsters.

Taken together, in the current study, using the hamster SARS-CoV2 model, we report that prophylactic intranasal treatment with Anu oil and til tailya reduced the lung viral load. However, SARS-CoV2–related pulmonary pathologies were prevented only in Anu oil–treated hamsters as demonstrated by histopathological lung injury scores and expression of injury markers and inflammatory cytokines. Although the chemical constituents of Anu oil and til tailya remain to be investigated. The protection against SARS-CoV2 infection and related pathologies seem to be in part due to the significant reduction in the viral entry and replication in the upper respiratory tract. This preclinical study in the hamster model points to the prophylactic potential of intranasal Anu oil in COVID and necessitates further studies to understand its observed effect.

## Data Availability

The original contributions presented in the study are included in the article/Supplementary Files; further inquiries can be directed to the corresponding authors.
